# Do Low Income Youth of Color See “*The Bigger Picture*” When Discussing Type 2 Diabetes: A Qualitative Evaluation of a Public Health Literacy Campaign

**DOI:** 10.3390/ijerph15050840

**Published:** 2018-04-24

**Authors:** Dean Schillinger, Jessica Tran, Sarah Fine

**Affiliations:** Department of Medicine and Center for Vulnerable, Populations University of California San Francisco, Zuckerberg San Francisco Hospital, 1001 Potrero Avenue San Francisco, CA 94118, USA; jessdqtran@gmail.com (J.T.); sarah.fine@ucsf.edu (S.F.)

**Keywords:** health literacy, social marketing, type 2 diabetes, diabetes prevention, qualitative research

## Abstract

As Type 2 diabetes spikes among minority and low-income youth, there is an urgent need to tackle the drivers of this preventable disease. *The Bigger Picture* (TBP) is a counter-marketing campaign using youth-created, spoken-word public service announcements (PSAs) to reframe the epidemic as a socio-environmental phenomenon requiring communal action, civic engagement and norm change. Methods: We examined whether and how TBP PSAs advance health literacy among low-income, minority youth. We showed nine PSAs, asking individuals open-ended questions via questionnaire, then facilitating a focus group to reflect upon the PSAs. Results: Questionnaire responses revealed a balance between individual vs. public health literacy. Some focused on individual responsibility and behaviors, while others described socio-environmental forces underlying risk. The focus group generated a preponderance of public health literacy responses, emphasizing future action. Striking sociopolitical themes emerged, reflecting tensions minority and low-income youth experience, such as entrapment vs. liberation. Conclusion: Our findings speak to the structural barriers and complexities underlying diabetes risk, and the ability of spoken word medium to make these challenges visible and motivate action. Practice Implications: Delivering TBP content to promote interactive reflection has potential to change behavioral norms and build capacity to confront the social, economic and structural factors that influence behaviors.

## 1. Introduction

Type 2 diabetes has drastically risen in the U.S. over the last decade, disproportionately affecting ethnic minority populations. The mean prevalence among adult minority populations with diabetes is 21.7%, in comparison to 11.3% among non-Hispanic whites [1]. Although Type 2 diabetes has historically been coined as “adult-onset” diabetes, it has increased by 30.5% in youth aged 10–19 years between 2001 and 2009 [2]. Among new cases of diabetes among youth, the vast majority of sub-types in white youth represent Type 1 (an autoimmune disease). In contrast, among minority youth, one–half to three-quarters of new cases represent Type 2 (a largely environmental disease) [3]. There is an urgent need to engage at-risk youth in preventing an illness that poses substantial risks for disabling complications.

Prevention efforts have primarily focused on individual behaviors and personal responsibility. However, encouraging youth to engage in healthy eating and physical activity is challenging. A limitation of previous approaches is an overemphasis on long-term health outcomes as the primary source of motivation. This focus on individual behavior change, despite the importance of the social, economic and environmental context in determining behaviors, reinforces the notion of “individual shame and blame” and contributes to a lack of effectiveness in engaging youth. Creating messages that align with values held closely by adolescents are much more likely to resonate with them and effect change [4]. Although adolescents might not be motivated by their health in the distant future, they are certainly not apathetic. While they are often regarded as being vulnerable to *hedonism* (focusing on pleasure and instant gratification), they do have strong drives towards *eudaimonism* (focusing on meaning and self-realization). Adolescents aspire to feel like socially conscious, autonomous people worthy of approval by their peers and others whose opinions they respect [4]. Such values include social justice, autonomy and defiance against authority. For example, the kinds of messages previously developed for the anti-tobacco Truth campaign—those that vilify the tobacco industry and its corporate henchmen and call out the targeting of minority consumers—represent attempts to tap into adolescent values of defiance against authority, the expression of autonomy, and social (in)justice. Moving adolescents towards healthier behaviors, such as not consuming soda or junk food, can become a way to “stick it to the man” and rebel against industry executives’ authority. In *The Bigger Picture* campaign (see below), minority youth poets have created content that enables youth to see the “the bigger picture”, e.g., the social and environmental forces that create and perpetuate diabetes, and motivates their peers to “take a stand against injustice”, eliciting “righteous anger and action” for social change [5,6]. 

A recent study of U.S. media coverage related to Type 2 diabetes revealed that only 12% mentioned social or environmental contributors; the vast majority focus on individual choices, unhealthy behaviors, or genetics [7]. *The Bigger Picture* (TBP, www.thebiggerpictureproject.org) differs from traditional diabetes prevention campaigns in that it is a counter-marketing, public health literacy campaign that harnesses minority youth-created spoken-word performance pieces produced as short films (public service announcements, PSAs). Counter-marketing campaigns use health communications strategies to reduce the demand for unhealthy products by exposing the motives of their producers and portraying their marketing activities as outside the boundaries of civilized corporate behavior.

TBP brings together the Arts with Public Health to create authentic and compelling content that speaks to and resonates with youth values of defiance against authority and the struggle for social justice. We have previously described the development and implementation of TBP [8]. While TBP process is curated by spoken word poet mentors and health communication experts, the creative work itself is generated by minority youth poets. TBP encourages young viewers to “take a step back” and observe and reflect on the larger social, structural and environmental forces that shape behavior and determine disease risk (Table 1). PSAs get disseminated via live high school assemblies and workshops, and social media, with the goal of increasing public health literacy and positively influencing behavioral norms [4,9].

*Individual health literacy* focuses solely on improving knowledge and capacity so the individual makes better health decisions [10], and consists of three dimensions: functional, interactive and critical. *Public health literacy* is “the degree to which individuals and groups can obtain, process, understand, evaluate, and act upon information needed to make public health decisions that benefit the community” [11]. This construct consists of three dimensions—conceptual foundations, critical skills and civic orientation—and differs from the construct of individual health literacy. Public health literacy contextualizes individuals as parts of a whole in the social ecology, engaging more stakeholders in advocating for community-level changes to address population health concerns. In combination, both individual and public health literacy provide a broader framework to promote health and reduce health disparities. Understanding the degree to which low-income youth integrate TBP as conveying individual vs. public health literacy messages will likely determine the effectiveness of the campaign. In addition, examining whether the context in which youth are exposed and respond to TBP messages (individual reflection vs. group-based reflection) has important implications as to how to best deliver, refine and amplify TBP messages to achieve the campaign’s goals.

## 2. Materials and Methods

### 2.1. Research Design

Since the focus of TBP is to shift discourse around Type 2 diabetes in youth away from individual “fault” or deficits, toward a more systemic, socio-ecological framework, we performed a non-experimental, qualitative study to examine whether and how TBP campaign PSAs advances health literacy among a sample of low-income, minority youth.

### 2.2. Sample

We recruited a convenience sample from *Youth Radio* (youthradio.org), identifying youth at the beginning of their Health Internship Program. *Youth Radio* is a non-profit media production organization in Oakland, CA that engages and trains low-income and minority youth in media literacy. In late 2014, the Program Coordinator asked her 13 health interns to participate in an interactive project on type 2 diabetes. The UCSF (University of California, San Francisco) study team offered these youth a $10 gift certificate to partake in a 1-h viewing of TBP PSAs, followed by a post-viewing questionnaire and a focus group discussion. The study was approved by the UCSF Institutional Review Board. We enrolled 10 of 13 potentially eligible youth, aged 15–20. They included 6 females and 4 males who were currently in high school or had completed high school education within the last 2 years. Six participants self-identified as African American, 2 as White, 1 as Mixed Race (Hispanic/Latino and African American), and 1 as Middle Eastern.

### 2.3. Intervention

We first delivered a 30-min non-facilitated presentation that included 9 representative TBP PSAs (average length 2:54 min, range 1:12–4:37). Genres of PSAs ranged from comedic parody to suspense; each PSA conveyed a central public health literacy message related to Type 2 diabetes. Table 1 lists the PSAs, including titles, genres, and intended public health messages. Table 1. Representative Bigger Picture Campaign Messages, Associated Adolescent Values, and Extent of PSAs’ Success in Conveying Messages.

### 2.4. Participant Responses to TBP Messages

Immediately after watching each PSA, participants completed a questionnaire with open-ended questions that asked participants, for each PSA, to reflect on: (a) what they felt the PSA was about; (b) what the biggest lesson of the PSA was; (c) what they liked or did not like about the PSA; (d) whether the PSA changed how they felt about Type 2 diabetes; and (e) what changes they would make to the PSA, if any. Because these questions were posed after each of the 9 videos, respondents were provided with up to 30 min of non-viewing time to complete the questionnaire.

The *Youth Radio* Coordinator, a Masters-level experience facilitator, then conducted a 1-h focus group that largely mirrored the survey questions, eliciting participant reflections with respect to specific and general opinions of the PSAs, understandings about Type 2 diabetes, and intentions to change behavior, including barriers to and facilitators of change. Moderator-guided facilitation provided a safe and confidential forum for participants to openly discuss their personal experiences and perspectives, maximizing opportunities to share a wide range of positive and negative opinions. During the focus group, brief video clips of all 9 PSAs were shown to participants as reminders to trigger conversation. The focus group was audio-recorded and transcribed.

### 2.5. Analysis

With respect to individual responses to each TBP PSA, we evaluated the degree to which participants integrated public health messages based on their responses to the questions: “What was the video about?” and “What was the biggest lesson?” Responses were grouped into one of four categories: participants fully integrated the PSA’s intended message; discussed a theme related, but not central, to the PSA’s intended message; expressed an unrelated message; or did not perceive a message. We also mapped responses into the broader constructs of *individual health literacy* and *public health literacy*, identifying salient themes within the relevant dimensions of each form of health literacy. 

Because we were interested in gaining insights into minority youth responses to the content of the PSAs from this public health communication campaign, the qualitative approach that we applied to the questionnaire and focus group data was exploratory and formative. Insofar as we did not have a specific hypothesis, we employed content analysis to identify emergent themes [12,13]. In a subsequent analytic phase, based on the overarching objective of the communication campaign and the patterns of youth responses that emerged, we mapped these themes onto distinct domains derived from the health literacy construct (see below). 

Members of the research team (JT and SF)—one a pre-doctoral student in Medicine and the other a Masters-level communication expert—independently coded the open-ended questionnaire responses and the transcripts to identify recurring themes. Iterative discussions amongst the study investigators, including the Principal Investigator (DS), a health communication scientist and public health physician, then led to a final set of overarching, predominant public health and sociological themes. Finally, based on the conceptual frameworks of Nutbeam [10] and Freedman [11], relevant coded statements were independently assigned by JT and SF into one of: (a) three dimensions of individual health literacy; or (b) three dimensions of public health literacy, respectively. Any disagreements as to the assignment of these statements were adjudicated by DS, who was blinded as to the source of the statements (e.g., individual response vs. focus group setting). 

We first coded the questionnaires and the focus group transcript line-by-line, categorizing responses by themes of the lowest complexity. We then organized the preliminary set of themes under overarching themes; to be considered an overarching theme, it needed to have at least 3 associated responses. Since the focus group discussion was often characterized by dualistic and competing perspectives on lived experiences as they relate to Type 2 diabetes, we also coded responses that reflected these tensions. Again, we mapped responses into the broader constructs of individual and public health literacy, identifying salient themes within health literacy dimensions. 

Finally, as PSAs can be viewed in vivo by individuals alone or in a facilitated group setting, we assessed differences in patterns of response across settings. To compare the individual vs. group setting responses, we organized coded statements pertaining to particular constructs and dimensions of health literacy in order of frequency, determined by counts of coded statements and calculated as a percentage of total coded statements.

## 3. Results

### 3.1. Participants’ Integration of Central Messages of PSAs from Post-Viewing Questionnaires

The percentage of participants who fully recognized a PSA’s intended public health message ranged from 22.2% to 70%, with the PSA entitled *Quantum Field* being the least and the PSA entitled *Sole Mate* being the most understood (Table 1). On average, more participants’ post-PSA questionnaire responses exhibited a complete understanding of the public health message (43%); a minority of participants expressed a theme related, but not central, to the public health message (15%) or described an unrelated public health message or no message (18%).

### 3.2. Prominent Public Health Themes

Four prominent themes related to factors associated with diabetes risk emerged from the initial coding framework of both individual and focus group responses: individual behaviors, built environment, financial barriers, and institutional factors. Below, we describe these themes; illustrative participant quotes can be found in Table 2.

Individual Behaviors. Proponents of this notion described how individuals have the choice to be healthy—to eat healthy and exercise—often assuming that they are in full control of their own health and well-being.

Built Environment. Some participants expressed understanding that Type 2 diabetes is not simply a result of individual behaviors and choices, but can also result from structural forces in the built environment [14]. Structural determinants included poor access to healthy food and drink options, e.g., the dearth of grocery stores and farmers’ markets vs. excess of corner stores and fast food establishments; unsafe neighborhoods; and insufficient recreational space for physical activity.

Financial Barriers and Competing Demands. In the context of poverty and food insecurity, individuals’ eating habits and choices are limited. Adults in low-income families are also often pressed for time, making cooking challenging. Therefore, some participants reported how youth often resort to high-caloric, nutrient-deficient food and beverages from the corner store for a fast “hunger fix” at a fraction of the price of healthier options.

Institutional Factors: Deceptive Marketing. Barriers at the institutional level involve deceptive marketing by profit-hungry food and beverage industries and lack of government regulation. Participants reported an understanding of how the food and beverage industry employs false advertising to sell unhealthy products to youth.

### 3.3. Underlying Sociological Themes

In addition to these “surface-level” themes, we identified three additional themes that reflected the underlying sociopolitical tensions faced by these youth when responding to the PSAs; these were more complex themes that likely reflected their lived experience. Below, we describe these themes; illustrative participant quotes can be found in Table 2.

Entrapment vs. Liberation. Some participants reported they felt confined by the structural barriers in their surroundings, conditioning them to lead unhealthy lives. In contrast, other participants aspired toward liberation from these entrapments through increased health literacy and social activation.

Powerlessness vs. Empowerment. While there was an appreciation for the need to address diabetes as a social justice issue, some participants reported a sense of futility and frustration. They believed that social action instigates change, but not always generates positive outcomes. These individuals felt that the epidemic has no fixable solution: the same corner stores will still be there and healthy food will not get cheaper anytime soon. On the other hand, other participants wanted to channel knowledge into action, either through intended personal or community-level change.

Cultural Determinism vs. Cultural Relativism. Some responses demonstrated a conflict between cultural determinism (wherein a dominant culture decides what is “right” or “wrong” in terms of health or nutritional value) and cultural relativism (wherein beliefs and customs have relative values within that individual’s social and cultural context). As such, some rejected PSA messages, articulating a rebellion against the dominant culture.

### 3.4. Differential Impact of Setting on Participant Responses

**Figure 1:**
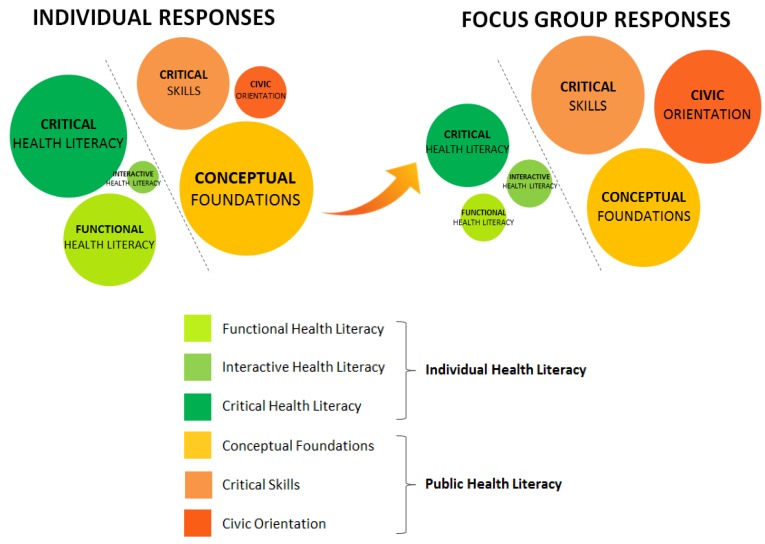
Distribution of Participant Responses in Individual vs. Focus Group Settings.

Figure 1 demonstrates the relative frequency of individual and public health literacy constructs emerging from questionnaire and group discussions. While there were more responses elicited in the individual setting than in the focus group setting, we observed a dramatic shift in content focus and emphasis between the two settings: from an individual health literacy construct (among individual responses) towards a public health literacy construct (among reflective group responses).

A total of 175 statements were coded from participants’ individual questionnaire responses. Seventy-nine of those statements (45.1%) were classified under *individual health literacy*: 29 out of 79 (16.6%) pertained to functional, 3 (1.7%) interactive, and 47 (26.9%) critical health literacy. The remaining 96 statements (54.9%) were classified under *public health literacy:* 56 (32.0%) conceptual foundations, 30 (17.1%) critical skills, and 10 (5.7%) civic orientation.

A total of 56 statements were coded from the focus group responses: 14 statements (25%) related to *individual health literacy*, with 3 statements (5.4%) related to functional, 3 (5.4%) interactive, and 8 (14.3%) critical health literacy. Forty-two (75%) responses were categorized under *public health literacy,* with 15 statements (26.8%) related to conceptual foundations, 15 (26.8%) critical skills, and 12 (21.4%) civic orientation.

Table 3 demonstrates the dimensions of health literacy within the two constructs of heath literacy (individual and public), as articulated by respondents in the individual and focus group settings. 

## 4. Discussion

In clinical settings, limited health literacy contributes to health disparities, especially among older adults, immigrants, racial/ethnic minorities, and low-income individuals [15]. Improving individual health literacy, therefore, is a promising strategy to improve population health, particularly in the management of Type 2 diabetes [16]. However, encouraging at-risk youth to prevent diabetes through traditional health education is often ineffective. Furthermore, such individual-level interventions do not support broader health promotion and health policy efforts to achieve public health goals. 

*The Bigger Picture* (TBP) is an innovative communication campaign that both features at-risk youth as creators and performers of novel public health content as well as targets at-risk youth. This model is relevant for conditions such as Type 2 diabetes, where exposures are determined by behavioral patterns solidified during adolescence. TBP model is unique in how it nurtures and supports the talent, authenticity and creativity of new health messengers: youth whose lived experience can be expressed in powerful ways. Never aiming to solely improve individual health literacy or direct individuals to change health behaviors, TBP attempts to harness core values of social justice and defiance against authority to improve youth public health literacy and foment action around social, environmental and policy change. While we are not aware of any research to shed light on the question as to whether defiance and social justice are more compelling for certain adolescent sub-groups, TBP campaign has yielded promising results in shifting low-income adolescent social norms [8]. We carried out this study to explore whether scaling up this model holds promise for low income and minority youth; determine whether the socio-ecological perspective, a complex and multilevel construct, can be integrated by youth exposed to TBP messages; and determine the extent to which facilitated discussions and reflective learning are needed to improve youth public health literacy. 

Our research found that TBP PSAs elicited responses that aligned with the broad constructs of both individual health literacy and public health literacy, and their respective dimensions. Individual responses after viewing PSAs revealed a fairly even distribution of statements representing individual and public health literacy. Immediately after viewing the PSAs, in the context of an open-ended questionnaire, some participants perceived TBP messages as conveying diabetes primarily as an individual concern; these participants reflected on intended personal changes such as eating “less sugar or processed foods” and “exercising more”. Other participants articulated a greater understanding of the broader social and environmental forces that shape individual behavior and can determine Type 2 diabetes risk, such as the lack of “affordable food, healthy food in poverty communities” and “how (companies) advertise the food, behind the scenes of the product”. These participants, moreover, proposed changes in their community through education and health promotion that suggest increases in public health literacy. 

In contrast, responses derived from the facilitated group discussions more heavily emphasized the roles of social, structural and environmental determinants, articulating a need to address diabetes through communal and civic engagement, demonstrating a more consistent paradigm shift toward public health literacy. In the setting of a group discussion, the PSAs served as vehicles for more extensive critical thinking, reflecting a more comprehensive public health literacy framework with a greater focus on socio-ecological constructs. This shift suggests that not only can a group setting amplify the messages to improve public health literacy in at-risk youth, but also supports the notion that TBP PSAs can align with youth values (defiance and social justice) to motivate social action and influence social norms.

The most striking themes that emerged in response to PSAs involved the tensions that these minority and low-income youth experience, expressed as sociopolitical themes such as entrapment vs. liberation. This speaks both to the structural barriers and behavioral complexities inherent to reducing diabetes risk in vulnerable communities, as well as the unique ability of the spoken word medium to make these challenges visible. Naming and reflecting on such tensions represent critical skills that raise communal consciousness and can promote civic engagement, fundamental dimensions of public health literacy. 

Our study has a number of limitations. First, the sample was based on the number of individuals involved in the Youth Radio internship. Due to limited funding and the size of the youth group that our partner, Youth Radio, had enrolled in its program, we were unable to carry out additional focus groups. As we now have created 27 video-poems, we are attempting to obtain funding for more youth focus groups. However, we are currently carrying out a randomized trial on Facebook to determine which framing messages most engage youth to view the video PSAs. Second, while ethnically diverse and of low income, the sample was likely not fully representative, as participants had self-selected to enroll in a media literacy program. However, we carried out this work at the beginning of their internship, making it less likely that exposure to the internship influenced their responses. Third, we cannot determine whether our comparisons of the impact of TBP messages across different communication settings (individual questionnaire responses vs. group responses) were a result of the setting and format, or the order in which we elicited responses. There certainly could be bias introduced by virtue of the order in which the videos were presented and the two ways in which the videos were discussed: (a) the first wave was after individuals viewed each one and responded as individuals; and (b) the second wave was in the context of discussing them in a focus group. Recall bias may favor the initial viewing; the focus group reflections reflect the aggregate impact of all videos. Relatedly, we cannot determine whether the shift to a more socio-ecologically oriented conceptualization in the group setting was a result of communication dynamics, or a result of participants integrating aggregate meta-messages across all nine PSAs. Fourth, participants viewed only 9 of the 27 current TBP PSAs, so we were not able to comprehensively evaluate all campaign messages. However, we selected the nine videos based on thematic, artistic and genre-related representativeness. Finally, no qualitative report can fully capture participants’ perspectives. For example, some participants revealed that they did not realize that TBP youth performers and their messages reflected authentic, first person narratives, assuming some were performed by youth actors reading from a script written by adults. Had our PSAs consistently made clear that low income and minority youth were delivering their own artistic interpretations of their lived experience, it is likely that PSAs’ impacts would have been even more robust.

This report suggests that TBP provides a promising artistic platform to communicate important factual and socio-political content related to the diabetes epidemic to low income and minority youth, one that appears to foster both individual and public health literacy. For some, the PSAs encouraged viewers to plan personal behavior changes and feel empowered to engage in community initiatives to prevent Type 2 diabetes. For others, the PSAs also revealed the structural and social barriers they face when trying to prevent diabetes—a revelation that was frustrating for some but activating for others. Our findings support the need for TBP—a campaign that focuses on the multilevel causes of diabetes—to be accompanied by interactive and action-oriented pedagogy if it is to achieve optimal impact. This interactive reflection could be facilitated via digital platforms or live group settings, and augmented by advocacy and action toolkits. We have recently expanded TBP’s digital reach, modernized the website and are curating a new TBP Facebook group that will allow for more interactivity. In addition, we have received seed funding to institute TBP programming in a more longitudinal fashion in public high schools, potentially enhancing public health literacy, enabling culture changes with respect to social norms, and promoting civic engagement. TBP process and content have also been harnessed by the local county health department to support public health action—specifically, the installation of fresh water stations in low-income neighborhoods that have the highest rates of consumption of sugar-sweetened beverages in the county. Finally, several community pediatric practices have reported using TBP content to motivate adolescents and families enrolled in their obesity or pre-diabetes clinics. These activities suggest that the model may have broad implications for public health, specifically related to an important social determinant of health: health literacy. 

## 5. Conclusions

TBP represents an innovative health communication and counter-marketing campaign that harnesses the talent and lived experience of minority youth poets who serve as messengers of novel and authentic first person content whose intent is to catalyze social action and influence social norms by aligning with adolescent values of defiance and social justice [5]. The key messages contained within TBP PSAs appear to often (but not always) hit home, frequently generating activating responses from youth for whom the messages are intended, both at an individual and communal level. TBP holds particular potential to promote diabetes-related public health literacy, thus building capacity among youth to both change behavioral norms as well as confront the social, economic and structural factors that largely determine these behaviors. It is likely that the TBP model is generalizable to other health conditions, and may appeal to youth stakeholders, not only to youth [6].

## Figures and Tables

**Table 1:** Representative Bigger Picture Campaign Messages ^a^, Associated Adolescent Values, and Extent of PSAs’ Success in Conveying Messages.

*The Bigger Picture* Campaign Spoken Word Piece and Film	Public Health Literacy Intended Public Health Message	Film Genre (and Accompanying Youth Value)	Participants Fully Understood the Film’s Public Health Message	Participants Discussed a Theme Related, But Not Central, to the Film’s Public Health Message	Participants Expressed an Unrelated Public Health Message	The Film Did Not Convey its Public Health Message
1. Pushin’ Weight	Profit-hungry food industries target youth with addictive sugary foods.	Dark Parody (Defiance)	3/10 Key Quote: “how from a young age sugar is shown as good and how fast it gets addictive but how bad it is for you”	4/10 Key Quote: “Sugar consumption”	2/10 Key Quote: “controlling your weight by watching what you eat”	1/10 Key Quote: “A drug dealer showing everyone how to sell drugs”
2. Product of His Environment	Institutionally reinforced social conditions, such as poverty, food insecurity, and violence, increase diabetes risk.	Drama (Social Justice)	6/10 Key Quote: “a young boy, how he doesn’t have access to food let alone healthy food so he starves and then only rarely can go to McDonalds so he is very unhealthy—and how messed up this cycle is”	2/10 Key Quote: “Not having enough money to provide healthy food”	1/10 Key Quote: “The biggest lesson is to eat right”	1/10 Key Quote: “About a black boy eating McDonalds and his father gets shot and he starts crying cause he broke and live in the hood”
3. Health Justice Manifesto	Policy call to action to address the Type 2 diabetes epidemic by challenging the government and corporations and advocating for the public’s health rights	Documentary/Anthem (Social Justice, Autonomy and Empowerment)	6/9 Key Quote: “About young people encouraging others to eat healthy and fight for health rights”One participant did not respond to questions pertaining to this video	1/9 Key Quote: “This video was about how liquor stores are in neighborhoods and grocery stores isn’t which makes people buy unhealthy food from liquor stores”	1/9 Key Quote(s): “It’s much more likely for young people to get diabetes these days”	1/9 Key Quote: “A knock off of a commercial I saw about not drinking at a party”
4. Block O’ Breakfast	Food and beverage industries utilize deceptive marketing and false advertisements to sell unhealthy, sugary and processed foods to young people.	Comedic Parody (Defiance)	3/10 Key Quote: “This videos was about how they advertise unhealthy food and that everything is not what it seems”	5/10 Key Quote(s): “How corporations pay the fee to be able to put all that junk in our neighborhood”	2/10 Key Quote: “bad breakfast that kids eat and what the effects can lead to”	0/10 N/A
5. Sole Mate	Prolonged, unmanaged Type 2 diabetes can lead to severe consequences, such as amputation of limbs. Increasing awareness can help prevent diabetes-related complications.	Horror (Social Justice)	7/10 Key Quote(s): “About diabetes causing amputation of body parts”	0/10 N/A	1/10 Key Quote(s): “stay healthy”	2/10 Key Quote: “How grateful we should be to be able to walk, but also to stop wars. There was too many messages”
6. Farm Livin’	We, as consumers, are clueless to what is happening behind the scenes of industrialized foods; we are being “fed” by profit-hungry corporations—like farm animals.	Documentary (Defiance)	3/9 Key Quote: “What people are eating, and what they do to the farm food for the consumers”One participant did not respond	3/9 Key Quote: “We don’t know what we eat or what we are putting in our body”	2/9 Key Quote: “We as consumers are just as bad as what we consume”	1/9 Key Quote: “About a rapper that’s chunky”
7. Death Recipe	Slavery and other forms of historical or contemporary forms of oppression shape dietary norms. Food addiction is a response to the stress and mental health problems that accompany oppression. Obesity and body image disorders are a result.	Autobiography/Testimonial (Social Justice and Defiance)	4/10 Key Quote: “How diabetes can be a part of culture or a family”	3/10 Key Quote: “a young lady who’s fed up about the way food is”	1/10 Key Quote: “how young people dying at a younger age from diabetes”	2/10 Key Quote: “not so clear”
8. Quantum Field	Trying to be healthy in an environment not conducive to healthy living feels like living in a nightmare.	Suspense (Defiance)	2/9 Key Quote: “our addiction to fast food is real. So much so, it’s odd when we wanna be healthy”One participant did not respond	1/9 Key Quote: “How it’s hard to escape diabetes… but possible”	1/9 Key Quote: “About unhealthy eating habits”	5/9 Key Quote: “that there’s good and bad people”
9. The Corner	Inaccessibility of healthy food options in low-income neighborhoods makes “choice” an illusion.	Testimonial (Social Justice and Autonomy)	3/8 Key Quote(s): “corner store convenience boost risks of diabetes”Two participants did not respond to questions pertaining to this video	1/8 Key Quote: “The dilemma between junk food and healthy food”	2/8 Key Quote: “all that unhealthy food is leading to diabetes”	2/8 Key Quote: “It was about a dude at a grocery store”

^a^ All films can be found at www.thebiggerpictureproject.org.

**Table 2:** Prominent Themes Emerging from Responses to Individual Questionnaires and Group Discussion and Illustrative Participant Quotes.

Primary Public Health Themes	Representative Quotes
Individual Behaviors	*“I can blame you for having diabetes, but you can win because it’s your way of living. That’s how you want to live because if you want to live like a hoarder, go ahead. If you want to live this way, it’s your choice to live. That’s why I say it’s within the individual”*
Built Environment	*“We know it’s the individual’s responsibility, but where restaurants are compared to where grocery stores is like strategically placed”*
	*“This video was about how liquor stores are in neighborhoods and grocery stores isn’t, which makes (our) people buy unhealthy food from liquor stores.”*
Financial Barriers and Competing Demands	*“These kids are hungry and they only go to certain places for food ‘cause that’s where they go. So if a kid only goes to the corner store because their parents don’t cook and there’s no grocery store close by, what are they supposed to do? They can go to a corner store for a $0.99 cent bag of chips… it’s convenient but it’s not good.”*
Institutional Factors: Deceptive Marketing	*“I mean it shows how I look at it… behind the scenes of the commercials of the food or… the advertisement of the food.”*
**Underlying Sociopolitical Themes**	**Representative Quotes**
Entrapment vs. Liberation	*“Even if people wanted to be healthy they don’t have the opportunity to go about it like financially or physically because they have nowhere to go.”*
	*“I want us to leave out of here with help and whatever we can.”*
Powerlessness vs. Empowerment	*“It’s… much bigger than our own so we can’t and—I hate to say it—that we can’t really do anything. We write as many letters to the government as we want, but they’re not going to take these liquor stores that have been here since I was a child. I’m sure somebody complained about them, they’re still here.”*
	*“I’m going to go tell somebody because it seems like—I didn’t used to know why the life expectancy of African American people was shorter that white people, but now that look at all the factors, it starting to make sense to me. So if you tell somebody else, maybe they want to eat healthier or something like that or maybe they have a better idea than me.”*
	*“(Let’s) start a garden in your community… (so) we have fresh produce in our garden. I mean we have in our garden, fresh produce I’d say that’s what I can think of like community-wise”*
Cultural Determinism vs. Cultural Relativism	*“There’s a lot of cultures that have things that Americans will look at and be like “Ugh! Why would you eat that?” but that’s their culture regardless if it’s healthy or not so it’s kind of for Black People, that’s our culture so, for you to say it’s unhealthy… It’s so offensive.”*
	*“I don’t like that… For the same reason, it was kind of tedious too and it’s like, “Okay, we know, Black people know.” That it’s not usually the healthiest thing to eat, but that’s culture.”*

**Table 3:** Representative coded rubric of responses related to dimensions of health literacy obtained in the post-film survey and focus group responses.

Dimensions of Health Literacy	Individual Health Literacy Dimensions of Individual Health Literacy (Nutbeam, 2006): Functional, Interactive, and Critical Health Literacy	Public Health Literacy Dimensions of Public Health Literacy (Freedman, 2009): Conceptual Foundations, Critical Skills, and Civic Orientation
**Conceptual Foundations**		Theme: **Built Environment (Please confirm whether the bold is necessary.)** “This video was about a boy that lives in a low income housing and had a unhealthy diet because there wasn’t grocery stores around” (2) Theme: **Institutional Factors: Deceptive Marketing** “How they [corporations] advertise the food, behind the scenes of the product” (4)
**Functional Health Literacy**	Theme: **Individual Behaviors** “The biggest lesson of this video was to show us how much sugar I eat” (1)	
**Interactive Health Literacy**	Theme: **Empowerment** “Should come together to change the way we eat” (3) Theme: **Liberation** “How it’s hard to escape diabetes…but possible” (8)	
**Critical Skills**	Theme: **Institutional Factors: Deceptive Marketing** “Be careful on what you buy because the things they say is in there is really not” (4)	Theme: **Liberation** “Do more research on the government and nutritions” (3) Theme: **Institutional Factors: Deceptive Marketing** “from a young age sugar is shown as good and how fast it gets addictive” (1)
**Civic Orientation**		Theme: **Empowerment** “we need to remove junk food places and put in more markets that sell cheaper healthier food” (2) Theme: **Financial Barriers and Competing Demands** “low income neighborhoods are at higher risk for diabetes due to the lack of resources” (2)
**Conceptual Foundations**		Theme: **Built Environment** “they can go to a corner store for a $0.99 cent bag of chips… it’s convenient but it’s not good, but they put it there” (10) Theme: **Institutional Factors: Deceptive Marketing** “you got to think about like a cartoon commercial… I feel like it was a parody… and then that’s how they were trying to market it to the kids, but it’s a company” (6)
**Functional Health Literacy**	Theme: **Individual Behaviors** “I feel like besides going to the store and buying food or like going in the McDonald’s, I feel like… you really want to lose weight and you’re concerned about what you eat then you should just like do it on your own like go walk or something. You walk or you don’t have to I mean I know it’s tempting, but if you really put your mind to it I feel like you could do it” (10–11)	
**Interactive Health Literacy**	Theme: **Empowerment** “in order to make a change within the community it has to start at a personal level. You can do community outreach things like that, but it might make a difference at that moment, but usually it doesn’t make a difference for long term like all these Treyvon Martin protests, they didn’t last for that long so nothing changed. Back at work, everyone’s back at school so nothing changed. So I think that if you really want to make a difference within your community, you have to start with you and maybe reach out to the people closest to you, reach out to people closest to them and that makes a chain reaction” (16–17) Theme: **Liberation** “I think that you should just say, ‘It’s hard to do but you still got to do it’” (9)	
**Critical Skills**	Theme: **Liberation** “once you listen to what he was saying, it was the facts. It made me not even want to mess none of that, growth hormone meat. None of that how fructose corn syrup” (8) Theme: **Built Environment** “for me living where I live, the closest healthy store is Trader Joe’s, but that’s across the bridge – that means I have to spend money to get there, I have to spend money when I get there so it’s hard to just be healthy. It’s not that easy” (9)	Theme: **Entrapment** “Even if people wanted to be healthy they don’t have the opportunity to go about it like financially or physically because they have nowhere to go” (9) Theme: **Entrapment** “after watching the videos, I think that to an extent, it’s a social justice issue for the reasons that Anonymous Number Two was saying…that the *restaurants are strategically put together* like for example, Hagen Burger, the shopping center by Wal-Mart, there’s “Wing Stop,” “In and Out,” “Candy Express,” “Chipotle,” “All-in-One,” and none of those are healthy at all. Yes, and then like Jamba Juice, they’re all together like there’s not one place there and then there’s a McDonald’s in Wal-Mart” (9)
**Civic Orientation**		Theme: **Empowerment** “Start a garden in your community. The center we have we have fresh produce in our garden. I mean we have in our garden, fresh produce I’d say that’s what I can think of like community-wise” (10) Theme: **Financial Barriers and Competing Demands** “so it’s like all these places put in one place and that area is not a place where rich people live. So it’s like - it’s kind of scandalous in a way” (9)
